# A Mobile Phone-Based Life Skills Training Program for Substance Use Prevention Among Adolescents: Pre-Post Study on the Acceptance and Potential Effectiveness of the Program, Ready4life

**DOI:** 10.2196/mhealth.8474

**Published:** 2017-10-04

**Authors:** Severin Haug, Raquel Paz Castro, Christian Meyer, Andreas Filler, Tobias Kowatsch, Michael P Schaub

**Affiliations:** ^1^ Swiss Research Institute for Public Health and Addiction at the University of Zurich Zurich University Zurich Switzerland; ^2^ Institute of Social Medicine and Prevention University of Greifswald Greifswald Germany; ^3^ Centre for Digital Health Interventions Institute of Technology Management University of St. Gallen St. Gallen Switzerland; ^4^ Energy Efficient Systems Group University of Bamberg Bamberg Germany

**Keywords:** coping skills, social skills, substance use disorder, adolescents, students, mobile phone

## Abstract

**Background:**

Substance use and misuse often first emerge during adolescence. Generic life skills training that is typically conducted within the school curriculum is effective at preventing the onset and escalation of substance use among adolescents. However, the dissemination of such programs is impeded by their large resource requirements in terms of personnel, money, and time. Life skills training provided via mobile phones might be a more economic and scalable approach, which additionally matches the lifestyle and communication habits of adolescents.

**Objective:**

The aim of this study was to test the acceptance and initial effectiveness of an individually tailored mobile phone–based life skills training program in vocational school students.

**Methods:**

The fully automated program, named *ready4life*, is based on social cognitive theory and addresses self-management skills, social skills, and substance use resistance skills. Program participants received up to 3 weekly text messages (short message service, SMS) over 6 months. Active program engagement was stimulated by interactive features such as quiz questions, message- and picture-contests, and integration of a friendly competition with prizes in which program users collected credits with each interaction. Generalized estimating equation (GEE) analyses were used to investigate for changes between baseline and 6-month follow-up in the following outcomes: perceived stress, self-management skills, social skills, at-risk alcohol use, tobacco smoking, and cannabis use.

**Results:**

The program was tested in 118 school classes at 13 vocational schools in Switzerland. A total of 1067 students who owned a mobile phone and were not regular cigarette smokers were invited to participate in the life skills program. Of these, 877 (82.19%, 877/1067; mean age=17.4 years, standard deviation [SD]=2.7; 58.3% females) participated in the program and the associated study. A total of 43 students (4.9%, 43/877) withdrew their program participation during the intervention period. The mean number of interactive program activities that participants engaged in was 15.5 (SD 13.3) out of a total of 39 possible activities. Follow-up assessments were completed by 436 of the 877 (49.7%) participants. GEE analyses revealed decreased perceived stress (odds ratio, OR=0.93; 95% CI 0.87-0.99; *P*=.03) and increases in several life skills addressed between baseline and the follow-up assessment. The proportion of adolescents with at-risk alcohol use declined from 20.2% at baseline to 15.5% at follow-up (OR 0.70, 95% CI 0.53-0.93; *P*=.01), whereas no significant changes were obtained for tobacco (OR 0.94, 95% CI 0.65-1.36; *P*=.76) or cannabis use (OR 0.91, 95% CI 0.67-1.24; *P*=.54).

**Conclusions:**

These results reveal high-level acceptance and promising effectiveness of this interventional approach, which could be easily and economically implemented. A reasonable next step would be to test the efficacy of this program within a controlled trial.

## Introduction

Several biological, psychological, and social transitions that occur during adolescence are essential for a young person’s later-life trajectory [1,2]. These transitions offer opportunities for them to gain skills to achieve greater autonomy from adults, build social connections with peers, develop a positive body image, and form a sense of identity. However, these transitions also facilitate exploration and risk taking at a stage when cognitive functions of the brain are not yet fully developed [3]. Shifts of emotional regulations and increased risky behaviors result in vulnerabilities for mental and substance use disorders, which constitute the biggest contributors to the health burden of 10- to 24-year-old individuals [4]. Substance use and the development of substance use disorders often first emerge during adolescence and co-occur with mental disorders [1].

The age of onset of substance use is similar across high-income countries, with increasing levels and frequency of use beginning in mid-adolescence, peaking during early adulthood [5]. According to the World Health Organization (WHO) World Mental Health Surveys [6], the interquartile range of the age-of-onset distributions is typically 14 to 21 years for alcohol, 15 to 21 years for tobacco, 16 to 22 years for cannabis, and 19 to 28 years for cocaine.

As most young people do not fulfill criteria for problematic or disordered use, prevention programs and early intervention should be the focus, rather than substance-related treatment measures. A recent systematic review of studies assessing the effectiveness of prevention, early intervention, and harm reduction in young people for tobacco, alcohol, and illicit drugs demonstrated the effectiveness of taxation, public consumption bans, advertising restrictions, and minimum legal age, as well as the potential effectiveness of preventative interventions that deliver life skills training in educational settings [7]. Schools are particularly suitable settings to reach adolescents with preventative interventions because of the ease of delivery and access to young people within compulsory secondary education [7].

A Cochrane review on school-based programs for the prevention of tobacco smoking [8] concluded that combined social competence and social influence interventions had a significant effect at 1 year and at longest follow-up, whereas a social influences program on its own, multimodal community-wide initiatives, and information-only interventions were found to be ineffective. Another Cochrane review on school-based prevention programs for alcohol misuse in young people [9] concluded that generic psychosocial and developmental prevention programs can be effective. However, the methodological quality of the trials included in the analysis was poor, and this did not allow for any quantitative pooling of data. A Cochrane review on school-based prevention of illicit drug use [10] concluded that programs based on a combination of social competence and social influence approaches were most promising, on average exhibiting small but consistent protective effects to prevent drug use.

According to the WHO, life skills are “abilities for adaptive and positive behavior that enable individuals to deal effectively with the demands and challenges of everyday life” [11]. The majority of the generic programs addressing social competences and social influences that were included in the aforementioned reviews were based on Bandura’s social learning theory [12], which hypothesizes that children and adolescents learn substance use by modeling, imitation, and reinforcement, influenced by individual cognitions, attitudes, and skills. Moreover, substance use susceptibility is increased by poor personal and social skills.

Generic life skills programs to prevent substance use, such as the IPSY (Information + Psychosocial Competence = Protection) program developed in Germany [13] or the ALERT [14] or LifeSkills Training [15] programs developed in the United States, typically combine training in self-management skills, social skills (eg, self-awareness, coping strategies, assertiveness, or communication skills), and substance use resistance skills (eg, resisting peer pressure to drink alcohol and recognizing and resisting media influences promoting cigarette smoking).

Although these life skills training programs were effective at preventing the onset of specific substances [8,13] or at decreasing problematic substance use [9], their implementation and dissemination in schools present serious challenges [16]. First, teachers and other professionals need the time, motivation, knowledge, and skills to deliver the program. Second, extensive resources—in terms of personnel, money, and time allocated to deliver substance use prevention—are required to prepare and administer such programs.

Electronically delivered interventions (eg, via computer, Internet, or mobile phone) have the potential to overcome the aforementioned obstacles that hinder successful program implementation and dissemination of life skills training in schools at a larger scale. Electronically delivered interventions have a wide reach at a low cost and offer the opportunity to automatically deliver individually tailored contents that can be accessed at any time and in any place [17]. Furthermore, electronically delivered interventions might be more appealing for adolescents because they can better ensure privacy and tailor contents to their needs.

Beyond traditional personal computers, a promising means of delivering prevention programs is to do so remotely through the use of mobile technologies. In Switzerland, as in most other developed countries, almost all (98%) adolescents between the ages of 12 and 19 years own a mobile phone, and 97% of these phones are smartphones [18]. Most adolescents are familiar with how to use mobile phones and typically use them on a daily basis for texting, taking pictures, playing games, and so on. Mobile phone–based interventions can provide almost constant support to users, relative to interventions that can only be accessed at specific times or locations; and they provide a discrete and confidential means of intervention delivery [19].

Several studies have underlined the potential and effectiveness of substance-specific mobile phone–based programs for early interventions in adolescents already consuming specific substances such as tobacco or alcohol [20-22]. However, the feasibility and effectiveness of more generic, life skills interventions to prevent substance use via mobile phones have not been addressed to date.

Consequently, the objectives of this study were (1) to test the acceptance, use, and evaluation of a mobile phone–based life skills program among vocational school students and (2) to explore its potential effectiveness.

## Methods

### Setting

In most European countries, vocational schools are postsecondary public schools that are analogous to American community colleges. They are a part of the dual educational system that combines apprenticeships in a business context and vocational training in a school context. Vocational schools provide general education and specific skills for each particular profession.

On the basis of data from the Swiss Federal Statistical Office, approximately half of all Swiss adolescents aged 16 to 19 years currently attend vocational schools [23], with the highest proportions among adolescents aged 17 years (males: 60%, females: 48%) and 18 years (males: 58%, females: 47%).

### Design and Procedures

A longitudinal pre-post study design with assessments at baseline and after program completion (month 6) was used to test the initial effectiveness of the program. Prevention specialists from branches of the Swiss Lung Association (Aargau, Basel-Land, Basel-Stadt, Berne, Vaud, and St Gallen), with particular training in the study and program to be delivered, arranged sessions lasting 30 min in participating vocational school classes during regular school lessons reserved for health education. Within this session, the students were informed about and invited to participate in a study testing innovative channels for the provision of health-related information and life skills. The students were informed by the prevention specialists about the study’s aims and assessments, reimbursement, and data protection. Students were also informed that they could withdraw from program participation at any time, simply by sending a short service message (SMS) expressing their request to stop the program.

The mobile phone–based program and its association with a friendly competition with prizes were described in detail by the prevention specialists. To ensure sufficient participation and, thus, representativeness of the sample [24], students were informed that they would also receive a small reward for participating in the study. Each student was provided with a tablet computer or used his or her mobile phone for the screening process to assess for study eligibility and for study registration and the baseline assessment. Inclusion criteria for this study were (1) a minimum age of 16 years and (2) possession of a mobile phone. After being screened for the inclusion criteria and giving informed consent, study participants were invited to choose a username and provide their mobile phone number.

On the basis of previous results on the efficacy of texting-based programs for smoking cessation [25,26], vocational school students who smoked cigarettes regularly (at least four cigarettes over the preceding month and at least one cigarette within the preceding week) received a program version combining smoking cessation support based on the MobileCoach Tobacco program [27] and strategies for stress management. As this program primarily focused on smoking cessation and did not include comprehensive life skills training, we excluded regular smokers from this study.

After they had given their informed consent, study participants completed a baseline assessment directly on their mobile phone or on the tablet computer that they had been provided with. They received additional questions that were necessary to tailor their intervention’s content. Subsequently, participants received individually tailored Web-based feedback directly on their mobile phone. Over the subsequent 6 months, they received individually tailored life skills training provided via mobile phone texting. All subjects were invited to complete a Web-based follow-up assessment after program completion 6 months after their enrollment in the study. For this, they received a text message (SMS) with a Web link to the Web-based assessment. Up to two text message–based reminders were sent to those who failed to complete the follow-up assessment upon initial request.

The study protocol was approved by the Ethics Committee in the Faculty of Philosophy at the University of Zurich, Switzerland (date of approval: June 24, 2016) and the trial conducted in compliance with the Declaration of Helsinki.

### The Intervention Program ready4life

#### Theoretical Background and Intervention Contents

The intervention elements of the program called *ready4life* are based on social cognitive theory [28,29]. This theory relies on social learning theory, as it was founded on principles of learning within the human social context [12], though it has also integrated several concepts from cognitive psychology. Key concepts of this theory that are incorporated within the Web-based and text messaging–based life skills program are (1) outcome expectations (ie, beliefs about the likelihood and impact of the consequences of behavioral choices), (2) self-efficacy (ie, beliefs about one’s personal ability to perform a desired behavior that could be stimulated; eg, by mastery, experience, or persuasion), (3) observational learning (ie, learning new behaviors via exposure to them through interpersonal or media displays; eg, through peer modeling), (4) facilitation (ie, providing strategies, tools, and resources that make new behaviors easier to perform), and (5) self-regulation (ie, controlling oneself via monitoring, goal setting, feedback, and self-instruction).

The contents of *ready4life* rely on proven and widely disseminated life skills programs such as IPSY [13], ALERT [14], and LifeSkills Training [15]. The program addresses (1) self-management skills, (2) social skills, and (3) substance use resistance skills. As recruitment for this study was conducted within the German- and French-speaking part of Switzerland, all intervention contents were available in both German and French.

#### Technological Background

The intervention program was developed using the MobileCoach system. Technical details of the system are described elsewhere [30,31]. The MobileCoach system is available as an open-source project. Password protection and Secure Sockets Layer encoding are used to ensure the privacy and safety of data transfer.

#### Individually Tailored Feedback

Individually tailored Web-based feedback was given immediately after subjects completed the Web-based baseline assessment during school classes using tablet computers or mobile phones. This feedback comprised five screens, which included textual and graphical feedback on the following: (1) stress in general; (2) the individual level of stress in various domains; (3) the individual level of stress compared with an age- and gender-specific reference group, based on the Perceived Stress Scale (PSS) [32] and on data derived from a survey among Swiss vocational school students [33]; and (4) individually applied and suggested strategies to cope with stress.

#### Text Messages

**Figure 1:**
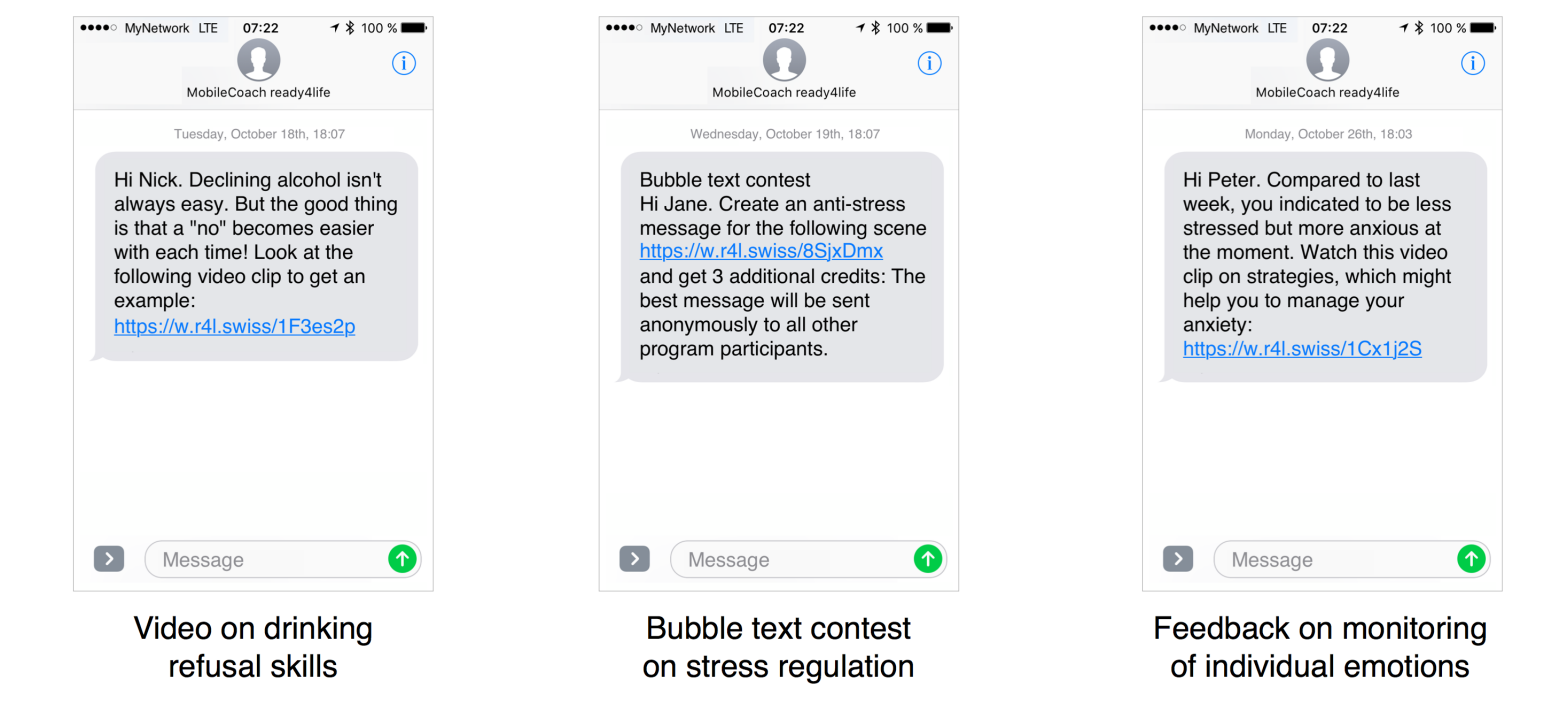
Sample messages (translated from the German program version).

For a period of 6 months, program participants received between two and four individualized text messages per week on their mobile phone. These messages were generated and sent by the fully automated MobileCoach system. Within the first 9 weeks, the messages focused on self-management skills; for example, coping with stress, emotional self-regulation, or management of feelings of anger and frustration. In the weeks 10 to 15, the messages focused on social skills, for example, making requests, refusing unreasonable requests, and meeting new people. In weeks 16 to 20, the text messages focused on substance use resistance skills, for example, recognizing and resisting media influences, social norms of substance use, or the associations of self-management skills and interpersonal competences with substance use. Boosters for each of the components were provided in weeks 22 to 24. The program concluded with information about a prize draw and an invitation to participate in the follow-up assessment. The messages were tailored according to the individual data from the baseline assessment and on-text messaging assessments during program runtime, for example, on substance use or the individual’s emotional state. Sample messages from different intervention components are displayed in Figure 1.

To exploit the full potential of current mobile phones, several interactive features—such as quiz questions, tasks to create individually tailored if-then behavior plans based on implementation intentions, and message contests—were implemented within the program. Within picture and message contests, participants were invited to create and upload text messages or photos on specific topics, for example, on individually preferred strategies to cope with stress or on relaxation possibilities. The messages or pictures provided by other participants could be rated anonymously on a separate responsive website by all participants, and the top three postings were presented anonymously to all other participants after 48 hours on a website, which was only accessible for program participants. All messages and photos created by the participants were checked by a junior scientist with respect to the appropriateness of their content. Inappropriate content was excluded and not presented to the other participants.

Due to the wide dissemination of mobile phones in adolescents [18], several messages also included hyperlinks to audio files (eg, audio testimonials and motivational podcasts), as well as thematically appropriate video clips, pictures, and related websites.

**Figure 2:**
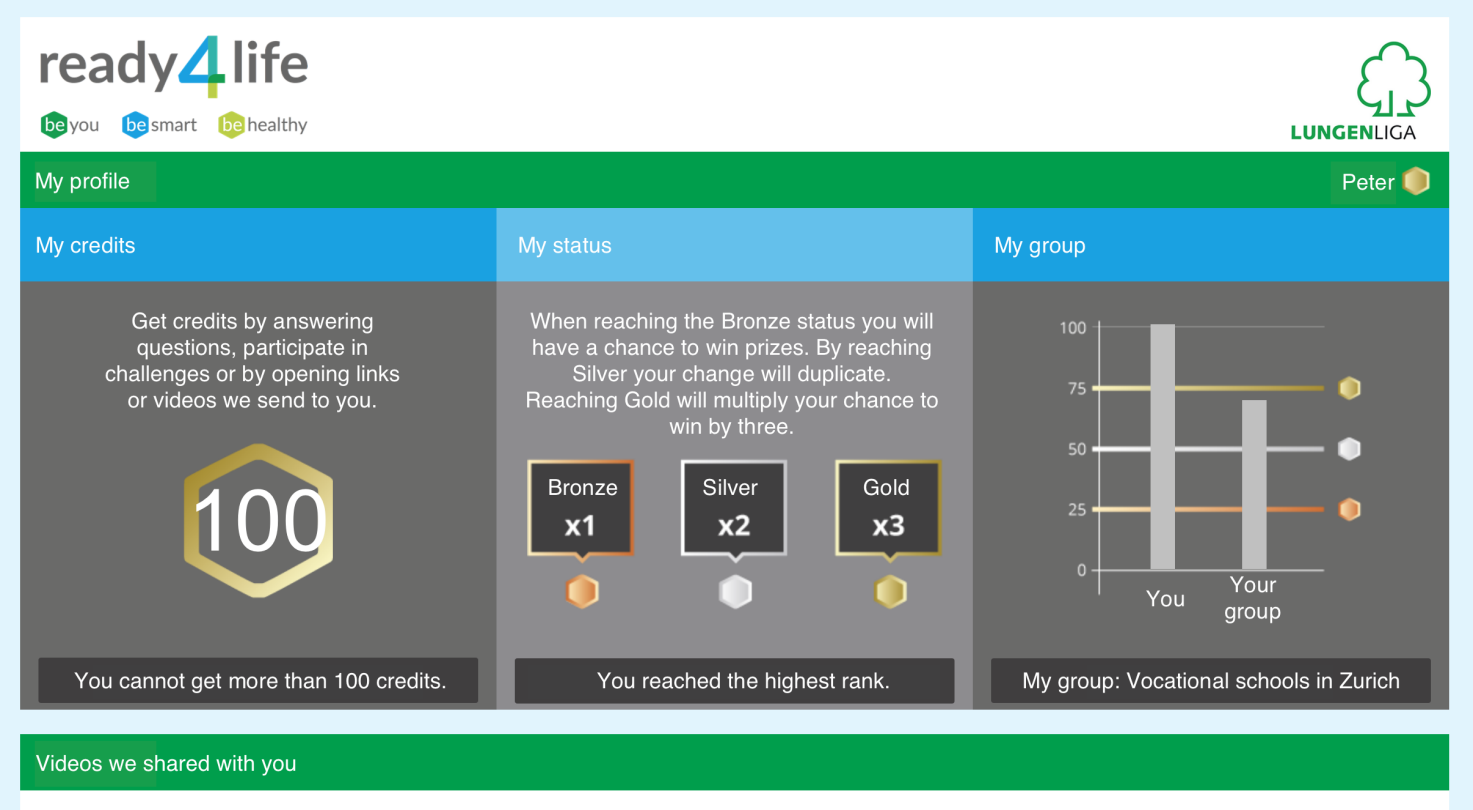
Individual profile page (translated from the German program version).

#### Prize Draw

To stimulate active program engagement, program use was associated with a friendly competition that allowed program users to collect credits for each interaction (eg, answering and monitoring text messages, participating in quizzes, creating messages or pictures within contests, and accessing video links integrated in text messages). The more credits participants collected, the higher were their chances of winning one of several attractive prizes that were part of a prize draw after program completion. Participants could retrieve their number of credits compared with the number of credits of other program participants of their group (similar starting date and Canton) at any time from an individual profile page (Figure 2).

#### Focus Groups for Pilot Testing

Before the study, a prototype of this program was tested and evaluated in five focus groups. Within these focus groups, individuals in the age range of 16 to 20 years, drawn from local vocational schools were asked to evaluate program flow, the layout and content of the Web-based assessment and feedback, and the content of text messages. Optimizations that resulted from these focus groups were integrated into the final version of the program.

### Measures and Outcome Criteria

#### Demographics

The baseline survey included questions about the following demographic variables: gender, age, and migrant background. For the last of these, we asked about the country of birth of both parents of each vocational school student to identify potential migrant backgrounds. On the basis of this information, persons were assigned to one of the following categories: (1) persons with neither parent born outside Switzerland—no migrant background and (2) persons with one or both parents born outside Switzerland—migrant background.

#### Life Skills

Life skills were assessed at baseline and 6-month follow-up. The life skills assessed were related to the contents of the program and focused on (1) stress, (2) self-management skills, and (3) social skills.

A four-item version [32] of the PSS [34] was used to measure the degree to which students appraised situations as stressful over the preceding month. Responses were scored on a 1- to 5-point scale from (1) “never” to (5) “very often.” These four items were “In the last month...,” (1) “how often have you felt that you were unable to control the important things in your life?” (2) “how often have you felt confident about your ability to handle your personal problems?” (3) “how often have you felt that things were going your way?” and (4) “how often have you felt difficulties were piling up so high that you could not overcome them?” This scale showed comparable acceptable psychometric properties when administered online and in paper-pencil format, with a Cronbach alpha of .72 for the Web-based version [32].

Self-management and coping behavior within vocational training were assessed using one item derived from each of the five subscales of the Questionnaire for the Measurement of Stress and Coping in Children and Adolescents (SSKJ 3-8) [35]. The items were selected based upon their item-subscale correlation and the relevance of their content for vocational school students. Students indicated, on a 5-point rating scale ranging from never (1) to always (5), how often they use each of the presented coping strategies in response to stressful situations during vocational training. These five items were “If I am stressed during vocational training...,” (1) “I tell others, how I feel” (seeking social support); (2) “I change something so that things are getting better” (problem solving); (3) “I tell myself that things will resolve themselves” (avoidant coping); (4) “I try to relax” (palliative emotion regulation); and (5) “I get totally upset” (anger-related emotion regulation). According to Eschenbeck et al [35], constructive coping behavior is particularly indicated by higher values on subscales (1), (2), and (4); conversely, higher values on subscales (3) and (5) are less desirable.

Social skills were assessed using a scale with seven items derived from the Assertion Inventory [36]. These items addressed (1) expressing an opinion that differs from that of other persons, (2) resisting social pressure to drink or smoke cigarettes, (3) telling a work colleague when he or she says or does something that bothers you, (4) asking questions to find out more about something, (5) apologizing when you are wrong, (6) accepting yourself even while being criticized, and (7) telling someone good news about yourself. Students indicated, on a 5-point rating scale ranging from never (1) to always (5), the frequency that they display each of the indicated behaviors. These seven items exhibited acceptable internal consistency, with a Cronbach alpha of .65.

#### Substance Use

Indicators of substance use were assessed at baseline and follow-up and included (1) at-risk alcohol use, (2) tobacco smoking, and (3) cannabis use.

At-risk alcohol use was assessed through the consumption items of the Alcohol Use Disorder Identification Test (AUDIT), the AUDIT-C [37]. The AUDIT-C assesses drinking quantity, drinking frequency, and binge drinking. On the basis of recommendations for adolescents [38], we used a cut-off value of ≥5 to determine whether risky drinking was present.

Tobacco smoking was assessed by the yes or no question: “have you taken at least one puff of a cigarette within the past 30 days?”

Cannabis use was assessed with the item “Within the last six months, how often did you use cannabis or marijuana?” with the response options (1) “never,” (2) “1-5 times,” (3) “6-20 times,” and (4) “more often than 20 times.” To estimate the prevalence of cannabis use within the last 6 months, we collapsed response options 2, 3, and 4 into a single category: cannabis use.

#### Program Use and Evaluation

To obtain the number of program participants who unsubscribed from the program within the program runtime of 6 months, we analyzed the log files of the MobileCoach system in which all incoming and outgoing text messages were recorded. Using these log files, we also assessed the mean number of replies to the 12 text message assessments during the program. At follow-up, we assessed another aspect of SMS usage by asking the participants whether they usually (1) read through the text messages thoroughly, (2) took only a short look at them, or (3) did not read the text messages.

Using a yes or no question, we evaluated whether the times when participants received the text messages were deemed to be appropriate. Furthermore, we assessed whether the number of received text messages was felt to be appropriate, or whether the participants would have preferred fewer or more messages. Finally, program participants were asked to rate the program and different program elements using the response categories “very good,” “good,” “less than good,” “bad,” and “don’t know.”

#### Outcome Criteria

To explore the intervention’s effectiveness, the pre-post changes between baseline and 6-months follow-up of the following variables were investigated: (1) perceived stress [32], (2) self-management and coping behaviors [35], (3) interpersonal skills [36], (4) at-risk alcohol use [37], (5) tobacco smoking, and (6) cannabis use.

### Data Analysis

To test for baseline differences between study participants and nonparticipants, Pearson χ^2^analysis for categorical variables and nonpaired student’s *t* tests for continuous variables were applied. For the attrition analysis (program participants lost to follow-up), we used χ^2^analysis for categorical variables and *t* tests for continuous variables. Baseline equivalence and lack of attrition bias were assumed for tests with *P*>.10.

We used generalized estimating equation (GEE) analyses to investigate the longitudinal course of the outcome criteria over the study period of 6 months. GEE is a repeated-measures regression model that takes into account the correlation of repeated measures within each subject [32]. It is a powerful and versatile procedure for analyzing longitudinal data, with minimal assumptions about time dependence, and it allowed us to use all available longitudinal data, irrespective of single missing values at follow-up.

We used logistic GEE models for binary outcomes and linear GEE models for continuous variable outcomes. To control for attrition bias, we additionally added the respective baseline variables and variables on program use as covariates to the GEE models. Each GEE model included (1) the examined time variable (baseline vs follow-up assessment) as a predictor, (2) covariates to account for selective attrition, and (3) some outcome variable as the dependent variable.

Given the clustered nature of the data (students within school classes and intraclass correlation for the considered outcomes ranged from .02-.06), we computed robust variance estimators for all GEE analyses. An alpha level of .05 (two-tailed) was chosen for all statistical tests conducted in the study. All analyses were performed using the Stata software package, version 12 (StataCorp).

## Results

### Study Participants

**Figure 3:**
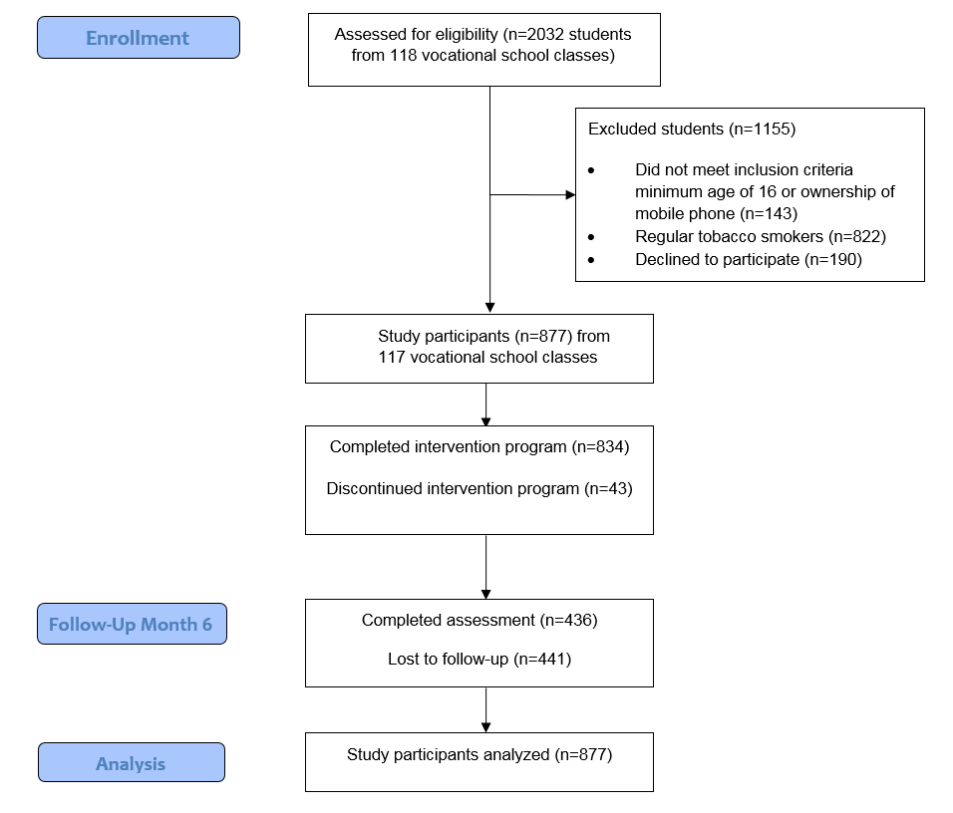
Participants’ progress through the study.

Participants’ progression through the study is depicted in Figure 3. At the time of the Web-based assessment in 118 vocational school classes at 13 Swiss vocational schools, a total of 2032 students were present. Among them, 1889 (92.96%, 1889/2032) had a minimum age of 16 years and owned a mobile phone and, as such, fulfilled the inclusion criteria for study participation. A total of 822 (43.51%, 822/1889) regular tobacco smokers were not considered within this study because they received a program focusing on smoking cessation. Among the 1067 (56.48%, 1067/1889) students who were invited to participate in *ready4life*, 877 (82.19%, 877/1067) agreed to participate. Nonparticipants were subjects who met enrollment criteria but did not agree to participate in the study.

Table 1 summarizes characteristics of study participants and nonparticipants. Study participants had a mean age of 17.4 years (standard deviation, SD 2.7) and consisted of 58.3% females. Study participants differed from nonparticipants with respect to the baseline variables *gender* and *migrant background*. A greater percentage of study participants than nonparticipants were female (χ^2^_1_=5.5, *P*=.02) and had no migrant background (χ^2^_1_=4.8, *P*=.03).

**Table 1:** Baseline characteristics of study participants and nonparticipants.

Variable		Study participants (N=877)	Nonparticipants (N=190)	*P* value
Female gender, n (%)		511 (58.3)	93 (48.9)	.02
Age in years, mean (SD^a^)		17.4 (2.7)	17.9 (4.5)	.10
**Migrant background, n (%)**				.03
	No migrant background	460 (52.5)	83 (43.7)	
	Migrant background	417 (47.5)	107 (56.3)	
Perceived stress (PSS^b^, range 1-5), mean (SD)		2.51 (0.66)	2.53 (0.64)	.65
**Self-management skills (range 1-5), mean (SD)**				
	Seeking social support	2.9 (1.3)	--	
	Problem solving	3.3 (1.0)	--	
	Avoidant coping	2.5 (1.1)	--	
	Palliative emotion regulation	3.3 (1.2)	--	
	Anger-related emotion regulation	2.6 (1.2)	--	
Social skills (scale, range 1-5), mean (SD)		3.7 (0.6)	--	
**Alcohol use (AUDIT-C^c^), n (%)**				.43
	Not at risk (<5)	710 (81.0)	128 (83.7)^d^	
	At risk (≥5)	167 (19.0)	25 (16.3)	
**Tobacco smoking in the previous 30 days, n (%)**				
	No	796 (90.8)	--	
	Yes	81 (9.2)	--	
**Cannabis use in the previous 6 months, n (%)**				.25
	No	769 (87.7)	129 (84.3)^d^	
	Yes	108 (12.3)	24 (15.7)	

^a^SD: standard deviation.

^b^PSS: Perceived Stress Scale.

^c^AUDIT-C: Alcohol Use Disorders Identification Test-C.

^d^n=37 missing values in nonparticipants.

Follow-up assessments were completed by 436 of the 877 (49.7%) study participants. Concerning attrition bias, the analysis revealed that follow-up assessments were completed more likely by female than male participants (χ^2^_1_=6.7, *P*<.01), by participants without a migrant background (χ^2^_1_=9.9, *P*<.01), by participants with higher values on the item on social support seeking (*t*_875_=−2.47, *P*=.01), and by participants with a higher number of program activities during the program period (*t*_875_=37.3, *P*<.001). To account for this attrition bias, these variables were entered as covariates within the GEE models that compared changes in outcomes between baseline and 6-month follow-up.

### Acceptability of the Intervention

During the program, which lasted for 6 months, 43 (4.9%) of the initial 877 program participants unsubscribed from the program, resulting in a total of 834 (95.1%, 834/877) participants who completed the entire program.

A total of 39 activities (eg, replies to texting prompts, accessing Web links within text messages, and participating in contests) were prompted over the 6-month program. The mean number of activities carried out by participants was 15.5 (SD 13.3). Of the 877 participants, 134 (15.3%) participated in no activities, 223 (25.4%) carried out 1 to 7 activities, 102 (11.6%) engaged in 8 to 14 activities, 82 (9.4%) in 15 to 21 activities, 123 (14.0%) in 22 to 28 activities, 156 (17.8%) in 29 to 35 activities, and 57 (6.5%) in 36 to 39 activities.

Of the 387 subjects with valid follow-up data, 323 (83.4%) indicated that they “read the SMS messages thoroughly,” 61 persons (15.8%) reported that they “took a short look at the feedback messages,” and only 3 persons (0.8%) chose the predefined response category “I did not read the feedback messages.”

The duration of the program was rated as appropriate by 334 (86.7%, 334/385) program participants with valid follow-up data. The number of received SMS messages was rated as appropriate by 84.9% (328/386); 6.7% (26/386) would have preferred more, and 8.3% (32/386) would have preferred fewer SMS messages. Almost all participants reported that the text messages were comprehensible (98.7%, 379/384). Participants were also asked whether the text messages were helpful, and 296 out of 384 (77.1%) agreed with this. Three out of 4 participants (73.2%, 281/384) indicated that they perceived the text messages as individually tailored to them.

**Figure 4:**
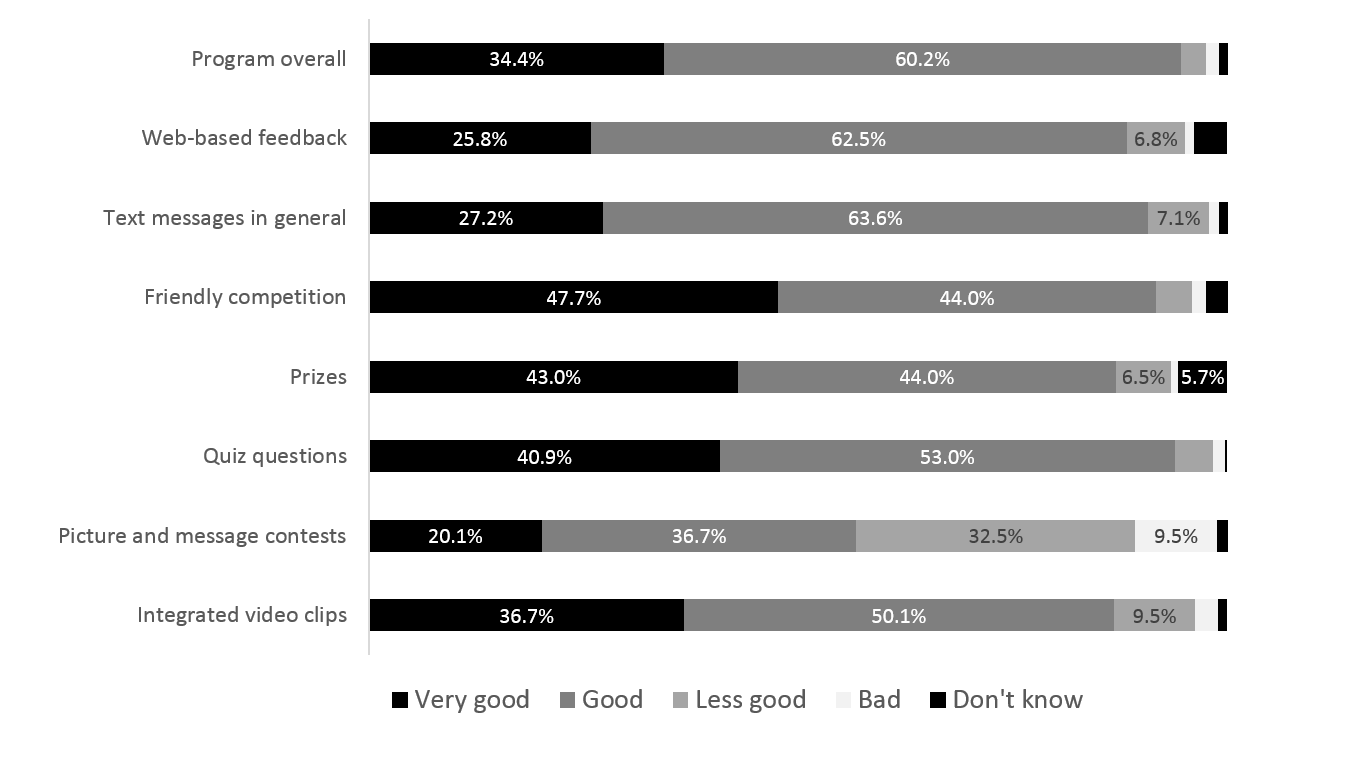
Evaluation of the program and specific program elements by program participants (n=384). Values are presented for percentages >5%.

Figure 4 presents additional evaluations of the program and specific program elements. The program overall was evaluated as “very good” or “good” by 94.6% of the participants. Out of the specific program elements, the competition for prizes, the quiz questions, and the text messages in general received the best evaluations, with >90% of participants rating them “good” or “very good.” The picture and message contests received the poorest ratings (56.8% “good” or “very good”).

### Program Effectiveness

#### Life Skills

Pre-post comparisons of the variables addressing life skills are displayed in Table 2. The GEE analyses revealed a statistically significant decrease in perceived stress (odds ratio, OR 0.93, 95% CI 0.87-0.99; *P*=.03). Meanwhile, statistically significant increases were obtained for the items addressing the self-management skills *seeking social support* (OR 1.18, 95% CI 1.05-1.33; *P*=.008) and *palliative emotion regulation* (OR 1.13, 95% CI 1.01-1.28; *P*=.04), as well as for the scale addressing social skills (OR 1.07, 95% CI 1.00-1.13; *P*=.04).

#### Substance Use

Pre-post comparisons of substance use prevalence rates are displayed in Table 3. Concerning alcohol use, GEE analyses revealed a statistically significant decrease in the percentage of persons with at-risk alcohol use from the baseline assessment to the follow-up assessment (OR 0.70, 95% CI 0.53-0.93; *P*=.01). No significant pre-post differences were obtained in the percentage of persons using cannabis or smoking cigarettes.

**Table 2:** Pre-post comparisons of variables addressing life skills.

Variable		Pre mean (SD^a^)	Post mean (SD)	OR^b^(95% CI)^c^ (N=877)	*P* value
Perceived stress (PSS^d^, range 1-5)		2.5 (0.7)	2.4 (0.7)	0.93 (0.87-0.99)	.03
**Self-management skills (range 1-5)**					
	Seeking social support	3.0 (1.2)	3.2 (1.2)	1.18 (1.05-1.33)	.008
	Problem-solving	3.4 (1.0)	3.3 (1.0)	0.93 (0.83-1.04)	.23
	Avoidant coping	2.4 (1.0)	2.5 (1.1)	1.09 (0.97-1.23)	.16
	Palliative emotion regulation	3.2 (1.2)	3.4 (1.1)	1.13 (1.01-1.28)	.04
	Anger-related emotion regulation	2.7 (1.2)	2.6 (1.2)	0.99 (0.87-1.11)	.81
Social skills (range 1-5)		3.8 (0.6)	3.9 (0.6)	1.07 (1.00-1.13)	.04

^a^SD: standard deviation.

^b^OR: odds ratio.

^c^Linear generalized estimation equation (GEE) models, with time variable (baseline vs follow-up assessment) as the predictor, adjusted for attrition bias.

^d^PSS: Perceived Stress Scale.

**Table 3:** Pre-post comparisons of variables addressing substance use.

Variable	Pre n (%)	Post n (%)	OR^a^(95% CI)^b^ (N=877)	*P* value
At-risk alcohol use, AUDIT-C^c^(N=420)	85 (20.2)	65 (15.5)	0.70 (0.53-0.93)	.01
Tobacco smoking, previous 30 days (N=392)	33 (8.4)	31 (7.9)	0.94 (0.65-1.36)	.76
Cannabis use, previous 6 months (N=419)	44 (10.5)	40 (9.5)	0.91 (0.67-1.24)	.54

^a^OR: odds ratio.

^b^Logistic generalized estimation equation (GEE) models, with time variable (baseline vs follow-up assessment) as predictor, adjusted for attrition bias.

^c^AUDIT-C: Alcohol Use Disorders Identification Test-C.

## Discussion

### Principal Findings

Within this study, we tested the acceptability and explored the potential effectiveness of a newly developed mobile phone–based life skills training program for substance use prevention among adolescents. The study revealed three main findings: (1) concerning participation, a large proportion of the eligible adolescents who were invited for program and study participation in the setting of a school classroom, participated; (2) concerning program use, the majority of program participants completed the entire program and engaged in program activities; however, regular program use could be improved; and (3) concerning program effectiveness, the initial results derived from this pre-post comparison revealed statistically significant increases in the life skills addressed, a decline in at-risk alcohol use, and stable prevalence rates for tobacco and cannabis use.

The proactive invitation for program and study participation in the school setting, in combination with the offer of a low-threshold mobile phone–based intervention, permitted us to reach 4 out of 5 adolescents for participation in the life skills program *ready4life*. Given the program duration of 6 months and that program participants needed to indicate their mobile phone number, this high participation rate is particularly remarkable and was even higher than for substance-specific mobile phone–based programs conducted in the same setting and using similar recruitment procedures; between 50% and 75% participated in comparable programs to support smoking cessation [25,26], whereas 75% participated in comparable programs to reduce problem drinking [39,40]. Beyond proactive recruitment in the school setting and during school hours, the following reasons might have contributed to the high participation rate we observed: (1) adolescents were invited by an institution independent of their school and teacher (anonymity); (2) the mobile phone–based program was flexible for use at any time and in any place, and withdrawal from the program was permitted at any time; (3) program participation and use were associated with participation in a friendly competition with the chance to win one of several attractive prizes; and (4) the program contents were developed specifically for adolescents during their vocational training.

Participation in the program was lower in male adolescents and among those reporting an immigrant background. These findings should be considered for program optimization, for example, by highlighting the relevance of this program for these subgroups or by emphasizing interesting program elements, focusing particularly on these target groups, during program presentations in school classrooms.

Overall acceptance of the intervention was good. Nearly all program participants (95%) stayed logged in until the end of the program, which lasted 6 months. The SMS messages were read by almost all program participants (94%), and 3 out of 4 participants reported that they were helpful and perceived the text messages as individually tailored to them. However, 15% failed to engage in any of the 39 program activities, and 52% engaged in fewer than half of the possible activities. On the basis of this finding, there is clearly room for improvement in terms of active program engagement, particularly concerning the picture and message contests, which received the poorest ratings among all program elements. The poor rating for this highly interactive element might be because of the limitations of mobile phone texting to receive and send pictures, which could be implemented more elegantly within a chat-based native mobile phone app.

The results concerning the initial effectiveness of this program derived from a pre-post investigation are promising. Data revealed a decrease in perceived stress, an increase in social skills, and increases in two out of the three desirable self-management strategies (seeking social support and palliative emotion regulation), whereas no changes were observed in less desirable self-management strategies (avoidant coping and anger-related emotion regulation). The proportion of adolescents with at-risk alcohol use was reduced by a quarter from baseline assessment to follow-up, whereas no significant changes were obtained in the prevalence of tobacco and cannabis use. On the basis of typically increasing levels and frequency of substance use in adolescence and early adulthood [41], these stable or decreasing prevalence rates might be attributable to program participation. However, no final conclusions on program effectiveness should be drawn from this study, as we could not control other factors such as fluctuations that might have occurred over the course of the year.

### Limitations

Beyond the limitations associated with the pre-post study design, some other study limitations should be mentioned. First, the results are restricted to adolescents without regular cigarette use, as only they were deemed eligible to participate in this general mobile phone–based life skills training. Second, only 50% of the study participants completed the follow-up assessment, which might have biased evaluations of the program and the results on efficacy, even though we controlled for attrition bias in our GEE models. Third, since the study focused on program appropriateness, meaning that we wanted it to be relevant to actual prevention practices, we restricted our outcome assessments to either short forms of, or single items extracted from more extensive validated instruments; furthermore all outcomes were self-reported.

### Conclusions and Outlook

This is the first study to test a comprehensive life skills training program for substance use prevention delivered by a mobile phone among adolescents. Our results suggest that this program, which delivers individualized messages and interactive activities integrated within a friendly competition, is both appropriate and promising in its effectiveness. Moreover, this intervention could be easily and economically implemented. On the basis of these initial positive results, a reasonable next step would be to test the efficacy of this program within a controlled trial.

Beyond testing the efficacy of digital-delivered life skills training programs, the examination of moderators and mediators of life skills trainings outcome in general remains an interesting question, which should be addressed in future studies.

Concerning moderators, it would be of particular interest to examine whether individuals with higher levels of substance use could also benefit from life skills training programs and to compare the effectiveness of these general life skills training programs with substance-specific interventions, for example, mobile phone–based programs for adolescents already consuming specific substances such as tobacco or alcohol [20-22]. Results from the IPSY program, conducted in young adolescents from Germany [13], indicated that this school-based and face-to-face delivered life skills training was ineffective for adolescents who are on a problematic developmental pathway of alcohol use. The authors conclude that this subgroup might be in need of an earlier, more intensive and tailored treatment compared with IPSY. In contrast, findings from the ALERT Plus project delivered in seventh and eighth grade, with booster lessons in the ninth grade, showed that curricula during high school can also be effective with at-risk youth, who are particularly likely to escalate drug use and experience drug-related harms [42].

Concerning mediators, it would be of particular interest to test which of the life skills addressed and successfully modified in turn might prevent or decrease problematic substance use, for example, results from the ALERT Plus project showed that program-induced changes in perceived social influences, one’s ability to resist those influences, and beliefs about the consequences of drug use mediated the effects on drug use [42].

Another interesting topic which should be addressed in future studies concerns potential spillover effects of general life skills training programs on other mental health conditions. As substance use and other mental disorders, for example, alcohol use disorders and depression, do not emerge as single impairments but rather co-occur [1,43], effects of these programs on further mental health–related outcomes should also be investigated. Concerning the comparison of substance-specific interventions and more general life skills training programs, one could assume that the latter might have an impact on a wider spectrum of mental health conditions.

## Abbreviations

AUDIT-C: Alcohol Use Disorder Identification Test-C
GEE: generalized estimating equation
IPSY: Information + Psychosocial Competence = Protection
OR: odds ratio
PSS: Perceived Stress Scale
SD: standard deviation
SMS: short message service
WHO: World Health Organization
